# Effects of lipopolysaccharide on maturation of bovine oocyte *in vitro* and its possible mechanisms

**DOI:** 10.18632/oncotarget.13965

**Published:** 2016-12-16

**Authors:** Shan-Jiang Zhao, Yun-Wei Pang, Xue-Ming Zhao, Wei-Hua Du, Hai-Sheng Hao, Hua-Bin Zhu

**Affiliations:** ^1^ Embryo Biotechnology and Reproduction Laboratory, Institute of Animal Science, Chinese Academy of Agricultural Sciences, Beijing 100193, China

**Keywords:** lipopolysaccharide, oocyte, cytoskeleton, oxidative stress, epigenetic modifications

## Abstract

Lipopolysaccharide disturbs the secretion of gonadotropin, endometrial function and implantation efficiency. However, there is little information regarding the effects of lipopolysaccharide on cyclic ovary activity, especially oocyte maturation. Therefore, we aimed to investigate the effects of lipopolysaccharide on the maturation potential of bovine oocytes. We found that lipopolysaccharide exposure significantly decreased the first polar body extrusion rate and delayed the cell cycle progression. The abnormal spindle rate was significantly increased in lipopolysaccharide treatment group, accompanied by disrupted localization and level of phosphorylated mitogen-activated protein kinase (p-MAPK). Moreover, lipopolysaccharide treatment significantly increased intracellular reactive oxygen species (ROS) levels and the early apoptotic rate in oocytes. The pro-apoptotic *caspase-3* and *Bax* mRNA levels and *caspase-3* protein level were significantly increased, whereas the anti-apoptotic *Bcl-2* and *XIAP* transcript abundance were significantly decreased in lipopolysaccharide exposure group. Furthermore, the dimethyl-histone H3 lysine 4 (H3K4me2) level was significantly increased, while the DNA methylation (5-mC) and dimethyl-histone H3 lysine 9 (H3K9me2) levels were markedly decreased in oocytes treated with lipopolysaccharide. In conclusion, lipopolysaccharide exposure inhibits the maturation potential of bovine oocytes by affecting cell cycle, cytoskeletal dynamics, oxidative stress, and epigenetic modifications.

## INTRODUCTION

Lipopolysaccharide is a main toxin from Gram-negative bacteria which cause mammary gland and/or upper genital tract infections such as mastitis and metritis, and impair the reproduction performance in females [1–3]. In bovine, nearly 80–90% of uteri were contaminated with bacteria after parturition, and the contamination may persist and manifest as subclinical or clinical metritis in 40% of animals [4–6]. This large scale postpartum metritis causes huge economic losses, contributing to female reproductive disorders which is estimated to cost $1.4 billion in the EU and $650 million in the United States annually [7, 8]. Lipopolysaccharide exposure also disturbs endometrial endothelial cell functions and causes sperm apoptosis in human [9, 10].

Recently, deleterious effects of lipopolysaccharide have been revealed, especially on reproductive performance. For instance, lipopolysaccharide could potentially suppress the secretion of gonadotropin-releasing hormone (GnRH) [11], reduce the concentration of circulating and intra-follicular oestradiol [12], extend the luteal phases [13], and decrease the conception rate [14]. After uterine infections, lipopolysaccharide was found to accumulate in the follicular fluid of growing follicles [12]. A recent study showed that lipopolysaccharide accumulation could reduce the primordial follicle pool, accelelate follicle atresia [15], and affect the oocyte developmental competence [16–18]. However, the mechanistic link between lipopolysaccharide exposure and the perturbation of follicles development or oocyte maturation remains to be explored.

Oocyte maturation, which is essential for the subsequent fertilization and embryo development, can be easily perturbed by a series of regulatory events such as oxidative stress, apoptosis and epigenetic modifications [19]. Oxidative stress can negatively affect spindle integrity and biological function [20], lower meiotic resumption rates [21], and then decrease oocyte maturation potential. Numerous studies have shown that lipopolysaccharide could induce cytotoxicity by enhancing cellular oxidative stress in different cell lines, including macrophage cell [22], dendritic cell [23], and epithelial cell [24]. The intracellular glutathione-dependent redox homeostasis could be altered by lipopolysaccharide exposure [22]. Moreover, Raza et al. reported that lipopolysaccharide could induces apoptosis by increasing the expression of oxidative stress-sensitive genes and proteins, and releasing the pro-apoptotic factor mitochondrial cytochrome c from the mitochondrial intermembrane space into the cytoplasm [25]. In addition, specific changes of histone H3 acetylation (H3-acetyl), dimethyl-histone H3 lysine 4 (H3K4me2), dimethyl-histone H3 lysine 9 (H3K9me2) levels occurred during lipopolysaccharide stimulation in human intestinal epithelial cells [26]. In oocyte, abnormal epigenetic modifications would lead to deficient chromosome condensation and segregation, and further delay the progression of maturation and trigger oocyte aging [27]. Studies on the toxic effects of lipopolysaccharide revealed that lipopolysaccharide could impair oocyte nuclear and cytoplasmic maturation, and decrease the proportion of cleaved embryos and the blastocyst formation rate after fertilization [17, 18]. However, the underlying mechanism by which lipopolysaccharide disrupts the maturation potential of oocyte has not been well elucidated.

The present study was conducted to assess the effects of lipopolysaccharide on the maturation potential of bovine oocytes and examine a preliminary exploration of the possible mechanisms involved through the aspects of cytoskeletal dynamics, oxidative stress, and epigenetic modifications.

## RESULTS

### Lipopolysaccharide exposure reduces the polar body extrusion rate of bovine oocytes

**Figure 1:**
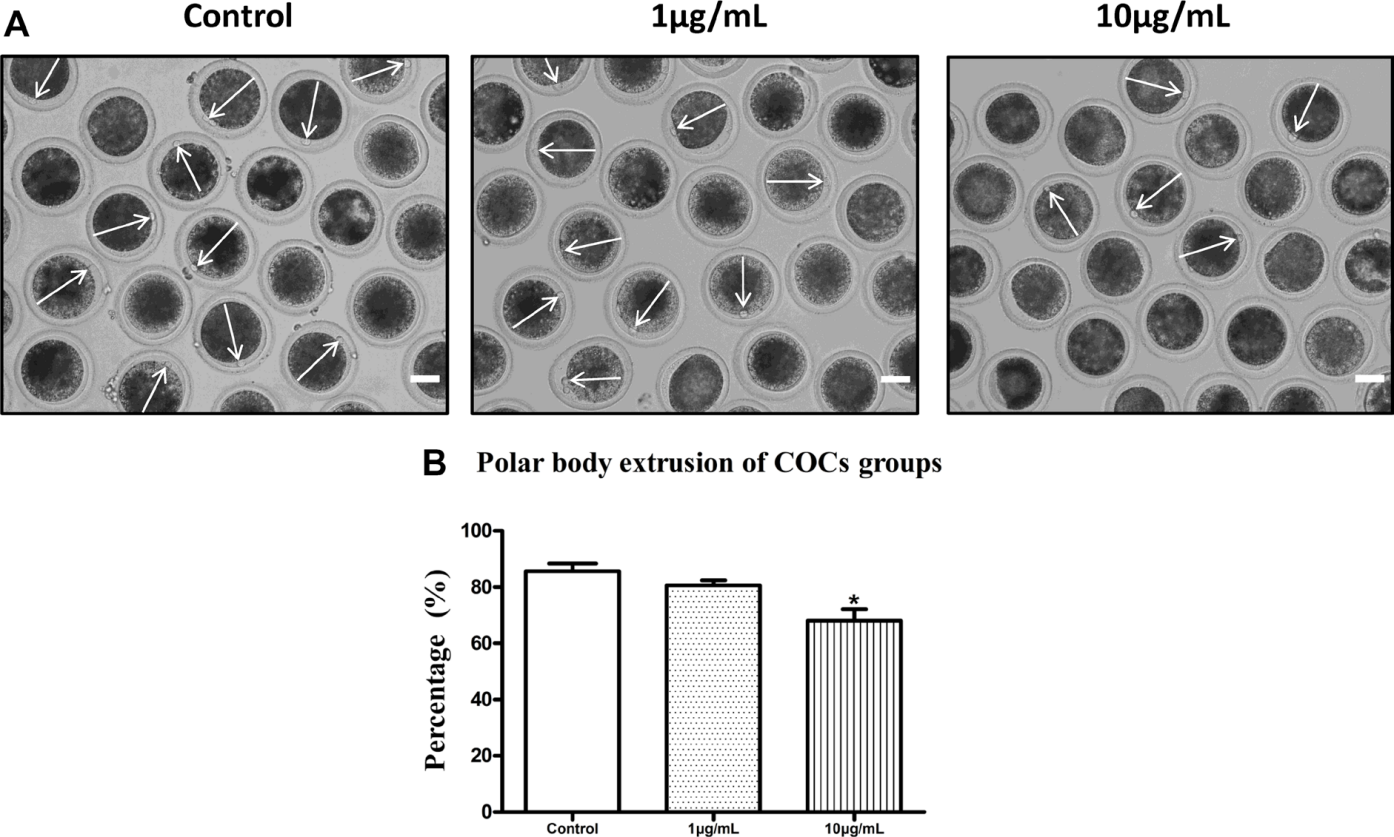
Lipopolysaccharide exposure reduced the polar body extrusion rate of bovine oocytes (**A**). Representative photomicrographs. The first polar bodies were indicated with arrows. Bar = 50 μm. (**B**). The polar body extrusion rate after lipopolysaccharide treatment. Asterisk indicates significant difference (*P* < 0.05).

The majority of oocytes in the control group were observed to extrude the first polar bodies after 22h of culture (85.67 ± 2.73%, *n* = 478). However, the polar body extrusion rates were suppressed after exposure to lipopolysaccharide (Figure 1A). Treatment with 1 μg/mL lipopolysaccharide resulted in a decrease in the percentage of first body extrusion rate, but no significant difference was observed between the treatment and the control group (80.60 ± 1.81%; *P* > 0.05; *n* = 205; Figure 1B). After treatment with 10 μg/mL lipopolysaccharide, the percentage of polar body extrusion rate was significantly reduced to 68.07± 4.09% (*P* < 0.05; *n* = 218) compared with the control group. Based on these results, we selected 10 μg/mL as the treatment concentration of lipopolysaccharide for subsequent experiments.

### Lipopolysaccharide exposure delays the cell cycle progression and affects spindle structure but not actin assembly in bovine oocytes

**Figure 2:**
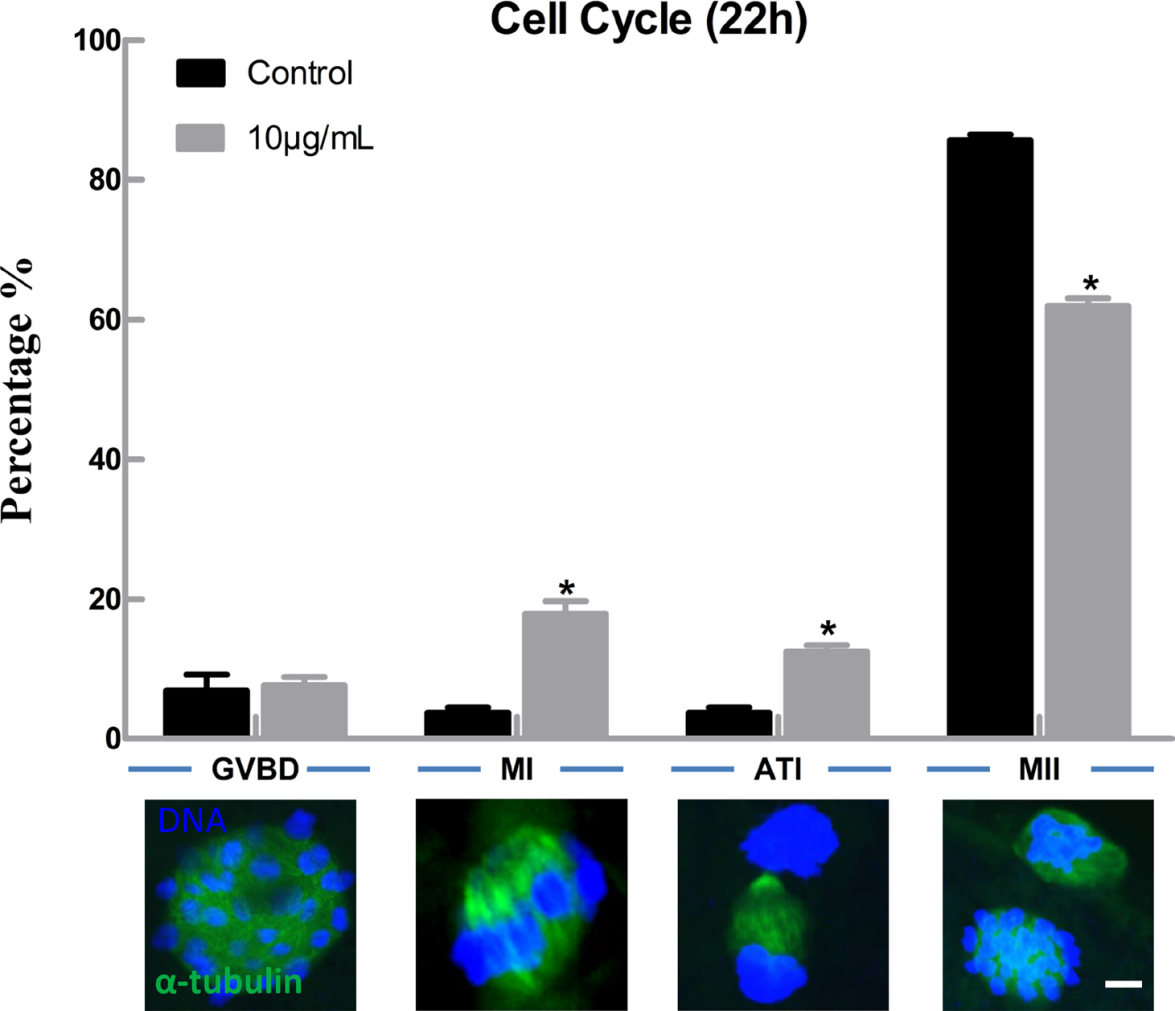
Lipopolysaccharide exposure delayed the cell cycle progression during oocyte maturation Rates of different stages of cell cycle after the oocytes were cultured for 22 h. Blue, chromatin; Green, α-tubulin. Bar = 1 μm. Asterisk indicates significant difference (*P* < 0.05).

In order to explore the possible reasons for decreased maturation rate of bovine oocytes after lipopolysaccharide treatment, we first analyzed the effect of lipopolysaccharide on cell cycle progression. The results showed that there was no significant difference in the proportion of germinal vesicle breakdown (GVBD) stage oocytes between the treatment (7.69 ± 1.15%, *n* = 133) and the control group (6.93 ± 1.31%, *n* = 125) (*P* > 0.05), but the percentages of oocytes arrested at metaphase I (MI) and anaphase and telophase I (AT1) stages were significantly increased (17.90 ± 1.81% and 12.46 ± 0.95%) compared to the control group (3.72 ± 0.44% and 3.72 ± 0.44% ) (*P* < 0.05; Figure 2).

**Figure 3:**
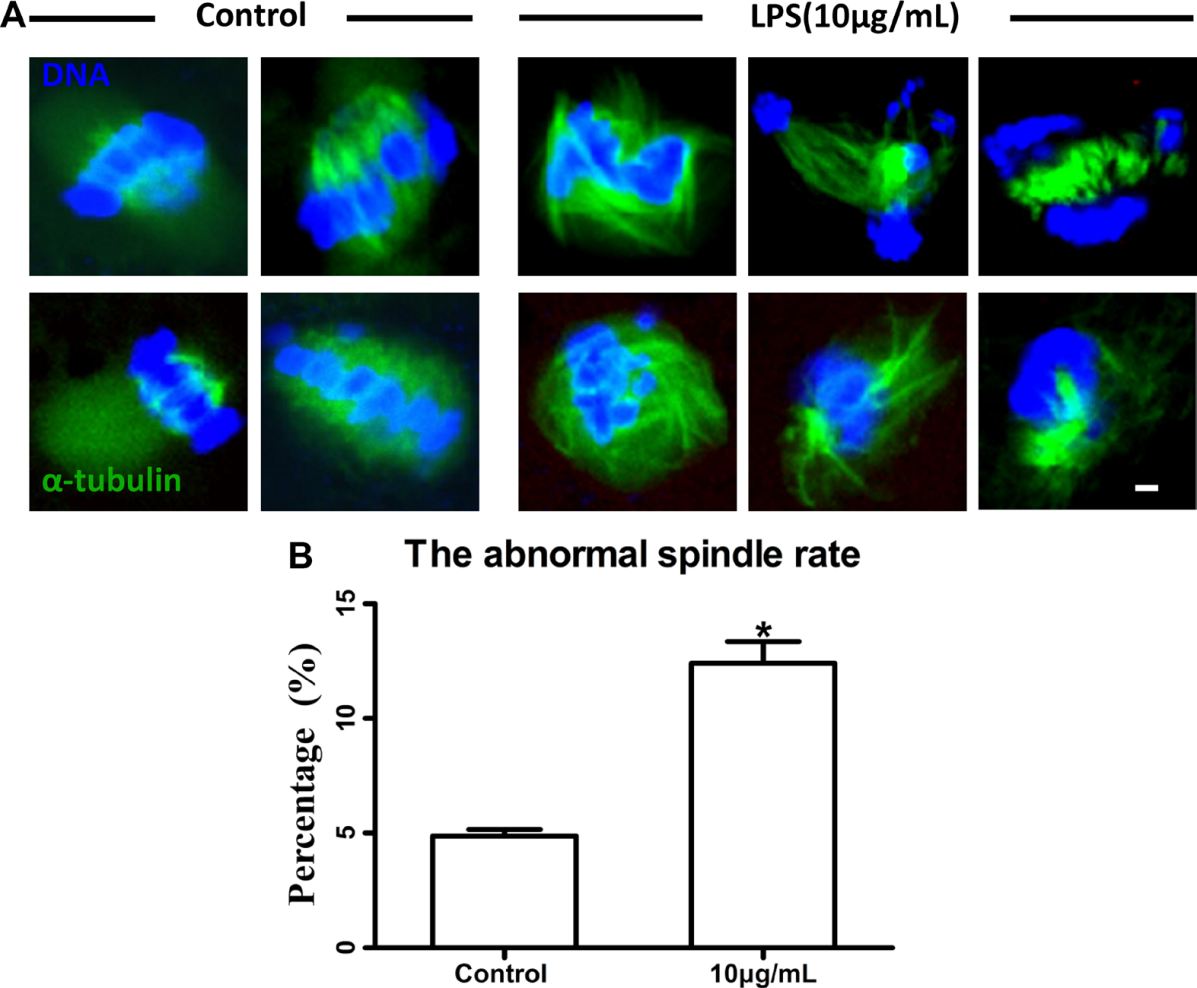
Lipopolysaccharide exposure impaired spindle structure (**A**). Representative photomicrographs. Blue, chromatin; Green, α-tubulin. Bar = 1 μm. (**B**). The abnormal spindle rate. Asterisk indicates significant difference (*P* < 0.05).

**Figure 4:**
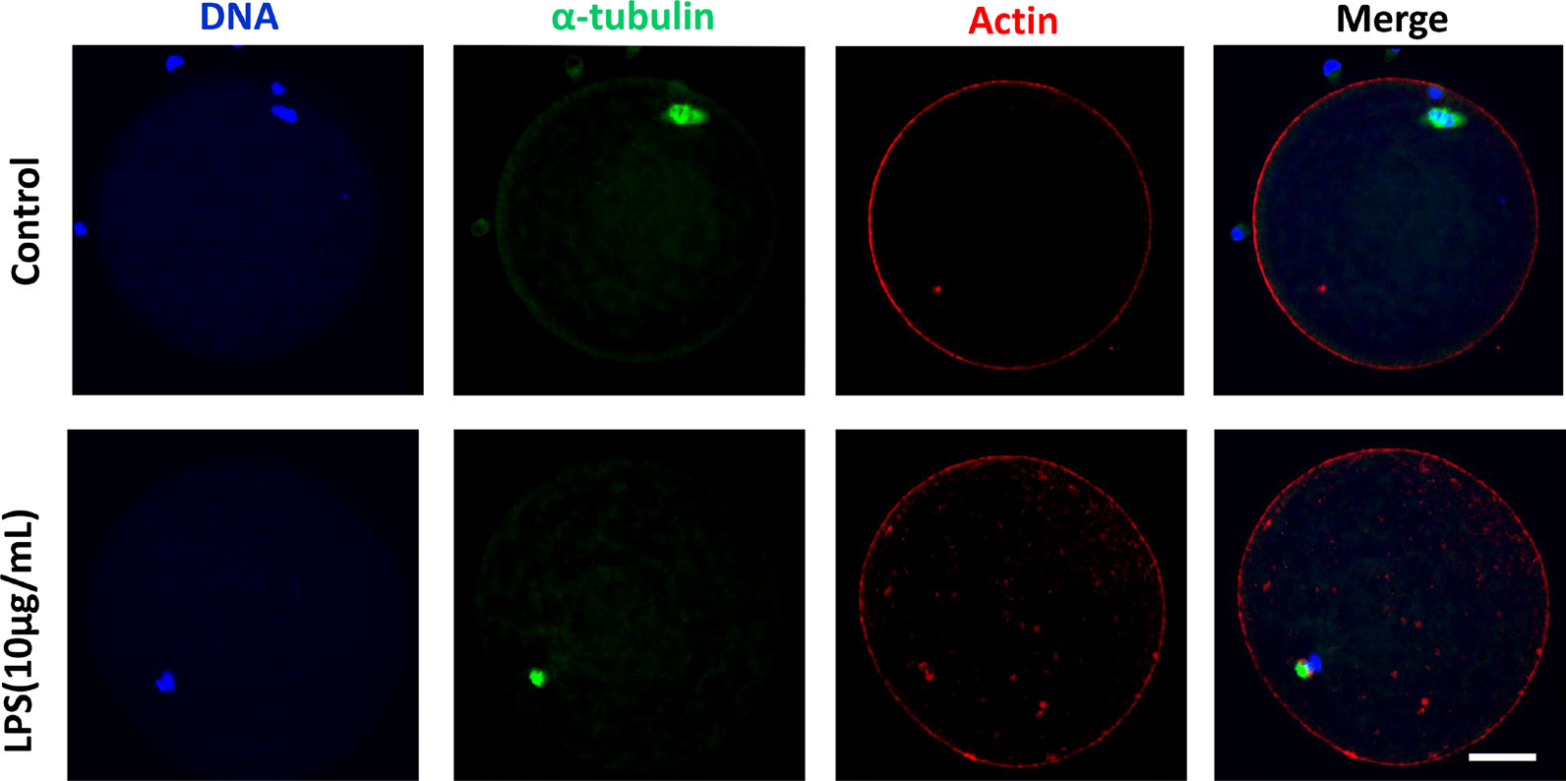
Actin assembly was not disrupted following lipopolysaccharide treatment The actin cap was present in both the treatment and the control groups. Blue, chromatin; Green, α-tubulin; Red, actin. Bar = 20 μm.

Following spindle morphology detection of MI oocytes exposed to lipopolysaccharide, we found that a substantial percentage of oocytes exhibited disrupted spindle morphologies and misaligned chromosomes. These oocytes had multiple poles, no poles, or disrupted poles (Figure 3A). In contrast, the majority of oocytes possessed normal spindle morphologies and well-aligned chromosomes in the control group. The percentage of abnormal spindle was significantly higher in lipopolysaccharide treatment group (12.40 ± 0.95%, *n* = 138) than that in the control group (4.87 ± 0.29%, *n* = 129, *P* < 0.05) (Figure 3B). The actin assembly was not affected by lipopolysaccharide exposure, as the actin cap was present in both the treatment and the control groups (Figure 4).

**Figure 5:**
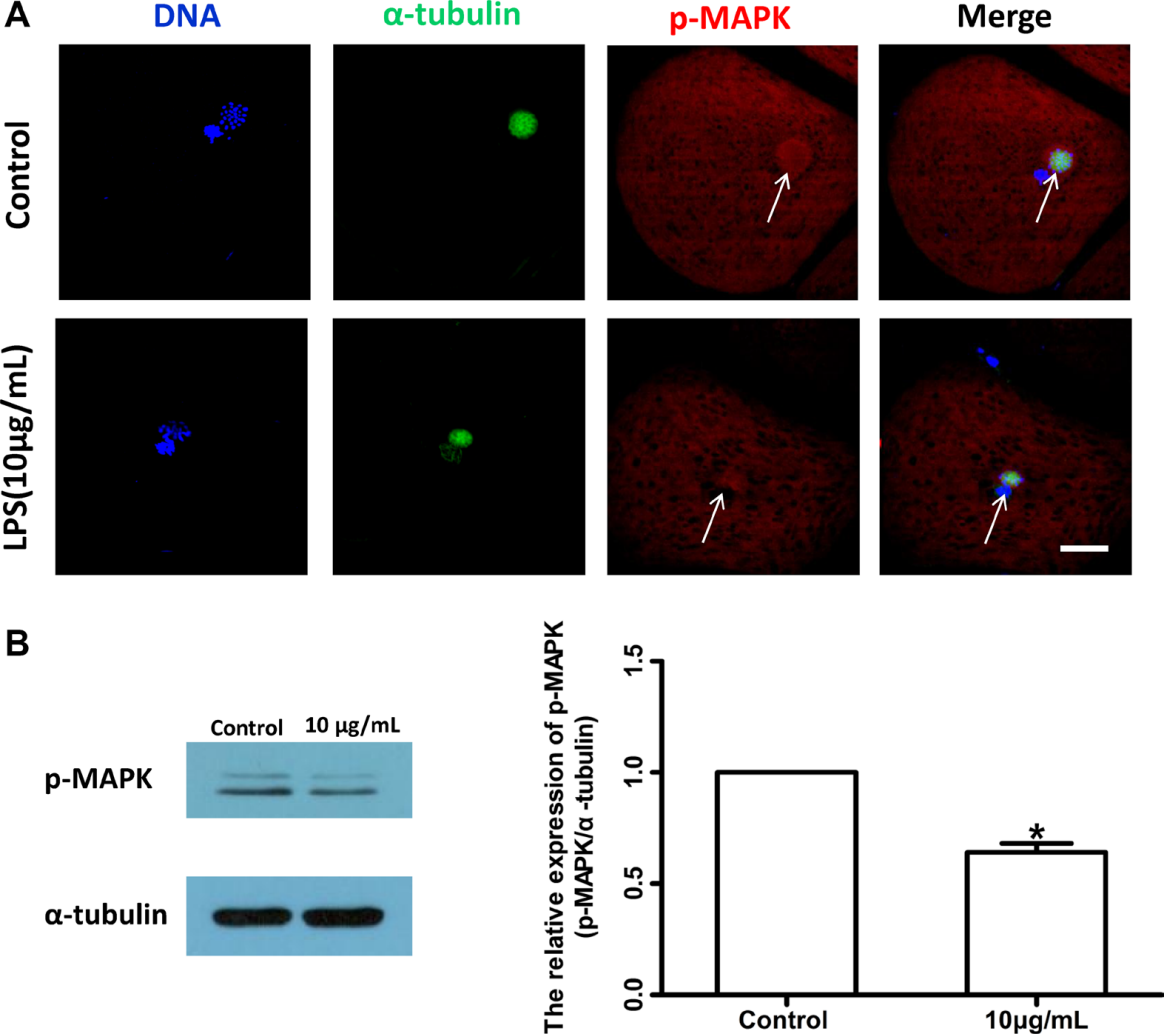
Lipopolysaccharide exposure changed the localization and protein level of p-MAPK in bovine oocytes (**A**). Localization of p-MAPK. Blue, chromatin; Green, α-tubulin; Red, p-MAPK. Bar = 20 μm. (**B**). Western blot analysis of p-MAPK level. Asterisk indicates significant difference (*P* < 0.05).

To further confirm the effects of lipopolysaccharide on meiotic spindle, the localization and level of phosphorylated mitogen-activated protein kinase (p-MAPK), a typical spindle regulator, were analyzed. As shown in Figure 5A, the localization of p-MAPK in oocyte exposed to lipopolysaccharide was disrupted (Control *n* = 47, Treatment *n* = 56), and the level of p-MAPK protein was significantly decreased compared with the control group (*P* < 0.05; Figure 5B). These results suggest that lipopolysaccharide exposure could delay cell cycle progression and disrupt the spindle structure of bovine oocytes.

### Lipopolysaccharide exposure induces oxidative stress and apoptosis in bovine oocytes

**Figure 6:**
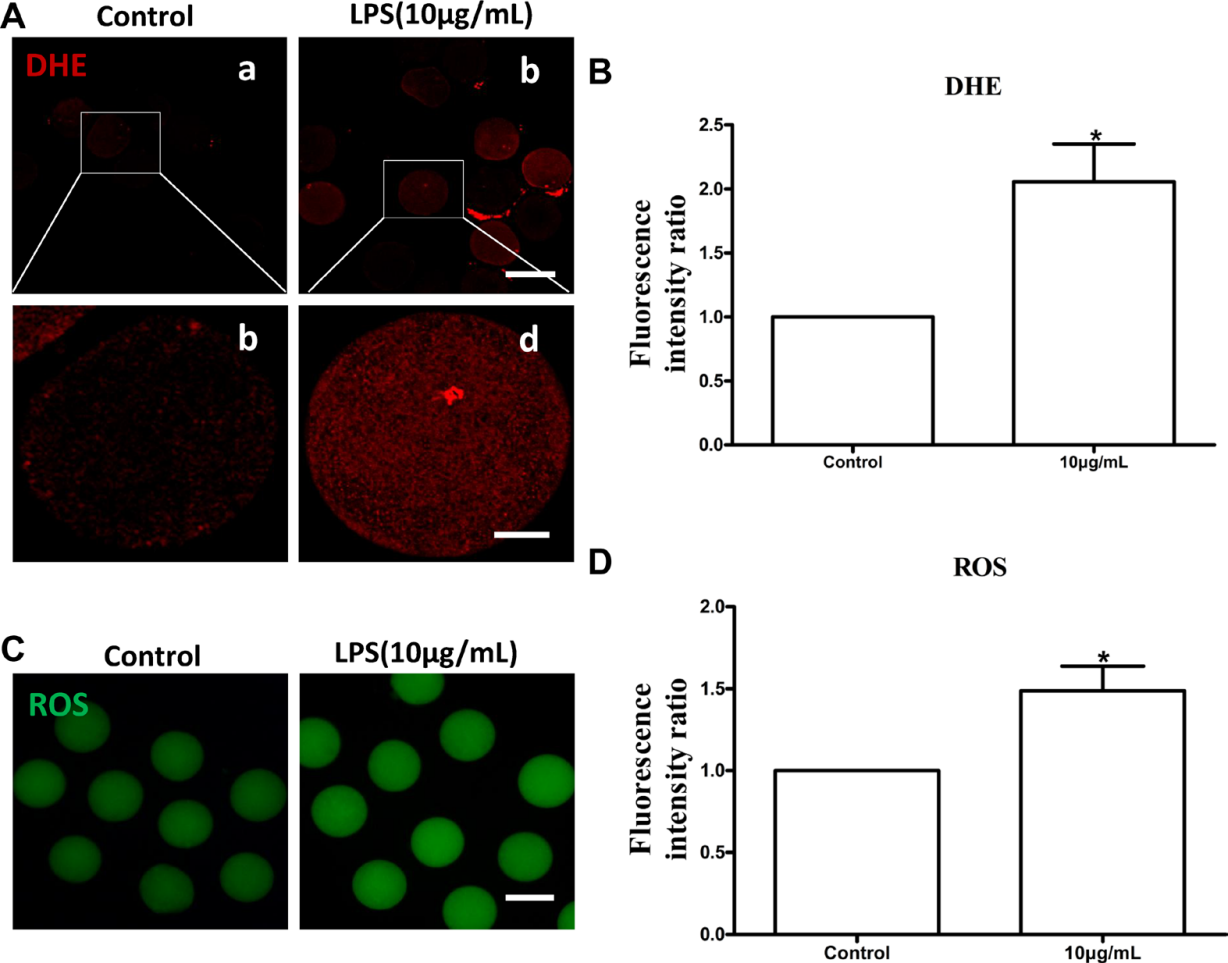
Lipopolysaccharide treatment increased DHE and ROS generation in bovine oocytes (**A**). Representative photomicrographs. Red, DHE. a, b: Bar = 100 μm; c, d: Bar = 20 μm. (**B**). The fluorescence intensity of DHE in lipopolysaccharide treated oocytes. (**C**). Representative photomicrographs. Green, ROS. Bar = 100 μm. (**D**) ROS generation after lipopolysaccharide treatment. Asterisk indicates significant difference (*P* < 0.05).

**Figure 7:**
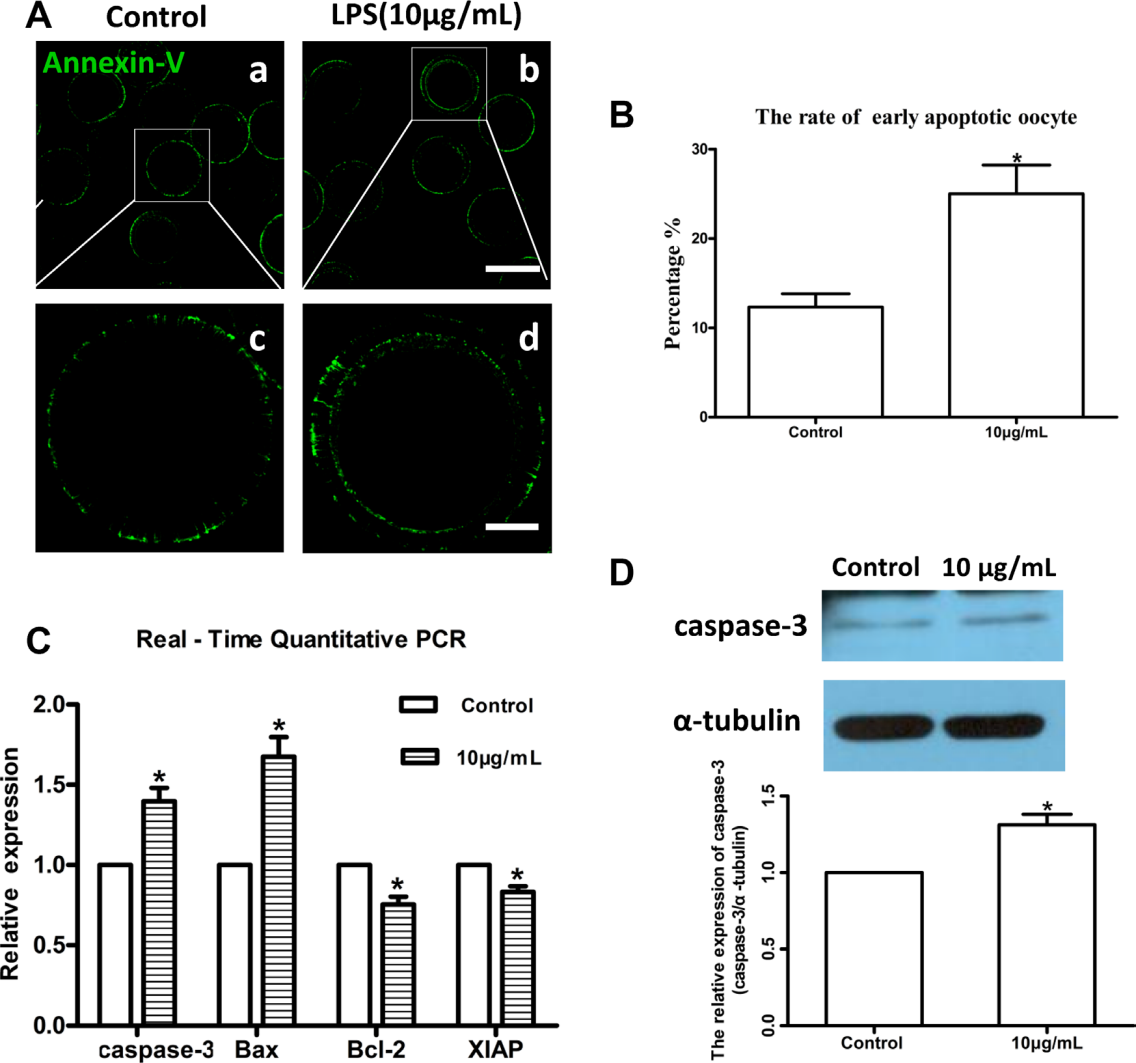
Lipopolysaccharide exposure induced apoptosis in bovine oocytes (**A**). Representative photomicrographs. Green, Annexin-V. a, b: Bar = 100 μm; c, d: Bar = 20 μm. (**B**). Rates of early apoptosis after lipopolysaccharide treatment. (**C**). Transcripts of apoptosis-related genes *caspase-3, Bax, Bcl-2*, and *XIAP*. (**D**). Western blot analysis of caspase-3 protein level. Asterisk indicates significant difference (*P* < 0.05).

Lipopolysaccharide treatment significantly increased intracellular dihydroethidium (DHE) and reactive oxygen species (ROS) levels in oocytes compared to the control group (*P* < 0.05; Figure 6B and 6D). These results indicated that lipopolysaccharide treatment induces oxidative stress in bovine oocytes. Annexin-V assay showed that the early apoptotic rate in oocytes exposed to lipopolysaccharide (25.00 ± 3.21%, *n* = 46) was significantly higher than that in the control group (12.33 ± 1.45%, *n* = 37; *P* < 0.05) (Figure 7A and 7B).

Moreover, the relative mRNA abundance of the pro-apoptotic genes *caspase-3* and *Bax*, and the anti-apoptotic genes *Bcl-2* and *XIAP* were determined by real-time quantitative PCR analysis. *Caspase-3* and *Bax* mRNA levels were markedly increased following lipopolysaccharide treatment, while *Bcl-2* and *XIAP* transcript abundances were significantly decreased compared to the control group (*P* < 0.05; Figure 7C). Western blot analysis further confirmed that caspase-3 protein level was significantly upregulated by lipopolysaccharide treatment compared to the control oocytes (*P* < 0.05; Figure 7D). Collectively, these results indicate that lipopolysaccharide could induce oxidative stress and apoptosis in bovine oocytes.

### Lipopolysaccharide exposure alters epigenetic modifications in bovine oocytes

**Figure 8:**
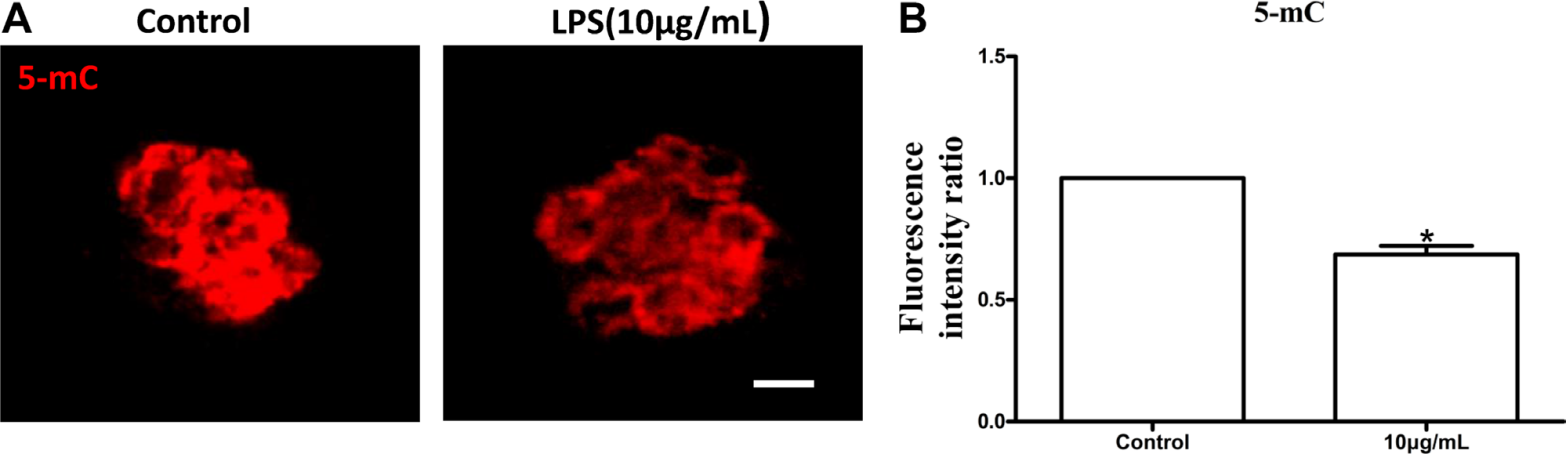
Lipopolysaccharide exposure decreased the levels of global DNA methylation (5mC) in bovine oocytes (**A**). Immunofluorescence staining for 5 mC in bovine oocytes. Red, 5mC. Bar = 20 μm. (**B**). The levels of global 5 mC in bovine oocytes. Asterisk indicates significant difference (*P* < 0.05).

**Figure 9:**
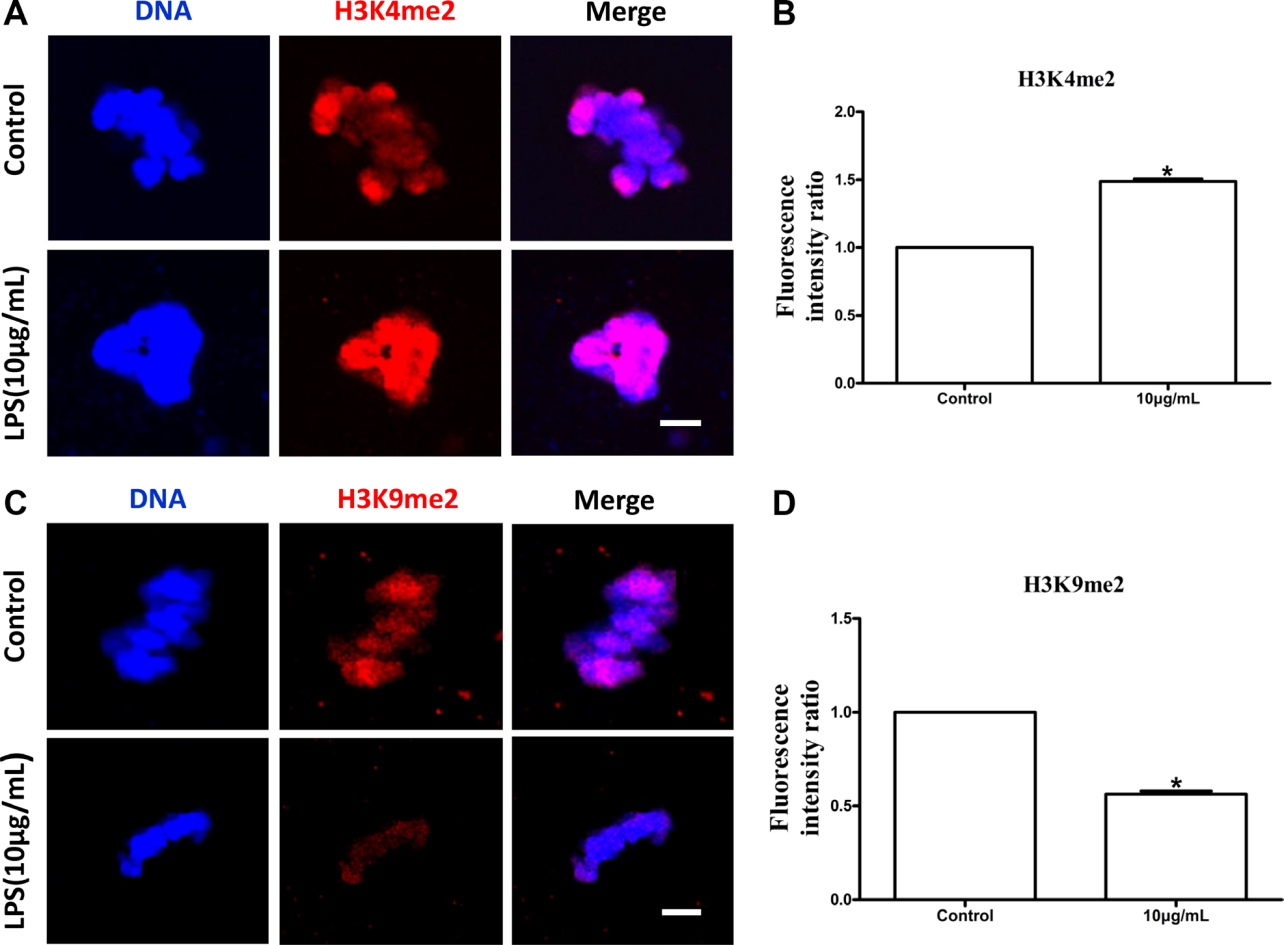
Lipopolysaccharide exposure changed the levels of global H3K4 methylation (H3K4me2) and H3K9 methylation (H3K9me2) in bovine oocytes (**A**). Immunofluorescence staining for H3K4me2 in bovine oocytes. Blue, chromatin; Red, H3K4me2. Bar = 20 μm. (**B**). H3K4me2 fluorescence intensity in bovine oocytes. (**C**). Immunofluorescence staining for H3K9me2 in bovine oocytes. Blue, chromatin; Red, H3K9me2. Bar = 20 μm. (**D**). Fluorescence intensity of H3K9me2 in bovine oocytes. Asterisk indicates significant difference (*P* < 0.05).

The levels of global DNA methylation (5mC), global H3K4me2 and H3K9me2 were measured in oocytes treated with lipopolysaccharide. Oocytes exposed to lipopolysaccharide presented lower 5mC levels than the control group (*P* < 0.05; Figure 8B). H3K9me2 levels in lipopolysaccharide treatment group were significantly decreased compared to control oocytes (*P* < 0.05; Figure 9D). In contrast, the global H3K4me2 levels in oocytes treated with lipopolysaccharide were higher than those in the control group (*P* < 0.05; Figure 9B). These data imply that lipopolysaccharide exposure may affect oocyte epigenetic status.

## DISCUSSION

In this study, we systematically analyzed the effects of lipopolysaccharide on cell cycle progression, meiotic spindle structure, oxidative stress, apoptosis, and epigenetic modifications in bovine oocytes matured *in vitro*. We found that lipopolysaccharide exposure delayed cell cycle progression, disrupted meiotic spindle structure, induced oxidative stress and apoptosis, and altered epigenetic status in bovine oocytes, which may provide possible reasons why lipopolysaccharide inhibited oocyte maturation potential.

Previous study showed that the proportions of oocytes that became blastocysts at day 8 after *in vitro* fertilization were significantly decreased after adding lipopolysaccharide to oocyte maturation medium [28]. Lipopolysaccharide exposure impaired oocyte nuclear and cytoplasmic maturation, and altered maternal gene expression in bovine [18]. In the present study, the first polar body extrusion rate was significantly reduced after treatment with 10 μg/mL lipopolysaccharide. This result is consistent with the report of Bromfield and Sheldon [16], who found that oocytes exposed to lipopolysaccharide had decreased polar body release rate. Moreover, we demonstrated for the first time that lipopolysaccharide exposure delayed cell cycle progression during the maturation process, as the proportion of oocytes arrested at MI and AT1 stages were significantly increased in lipopolysaccharide exposure group. Previous reports have shown that lipopolysaccharide could block cell cycle progression in human bone marrow-derived macrophages, microglia and B cells [29–31].

Dynamic changes in cytoskeleton are crucial for meiotic maturation and fertilization of mammalian oocyte. Lipopolysaccharide could induce meiotic failure, and perturb meiotic structures such as aberrant spindles, chromosomal ejection, or GVBD failure in *in vitro* matured cumulus-oocyte complexes (COCs). Our results showed that oocytes treated with lipopolysaccharide exhibited disrupted spindle bipolarity and structure, while the actin cap was not affected. Furthermore, lipopolysaccharide exposure decreased the protein level of p-MAPK. Microtubules form the bipolar spindle, which aligns the chromosomes on the metaphase plate and segregates them into the two daughter cells [32, 33], and actin filaments are responsible for the spindle movement [34]. As a regulator, MAPK plays a key role in microtubule organization and meiotic spindle assembly [35]. Thus we speculated that lipopolysaccharide perturbed spindle formation by suppressing p-MAPK level, resulting in the reduction of polar body extrusion rate of bovine oocytes.

Excessive generation of ROS originated from external surroundings and/or normal cell metabolism may lead to oxidative stress and further induce meiotic cell cycle arrest and apoptosis in mammalian oocytes or pre-implantation embryos [36, 37]. A number of studies showed that lipopolysaccharide could induce excessive ROS generation and apoptosis events in different cell lines [22, 23, 38, 39]. As expected, we found that lipopolysaccharide treatment significantly increased intracellular ROS levels and the early apoptotic rate in bovine oocytes. Moreover, the transcript abundance of pro-apoptotic *Bax* and *caspase-3*, as well as the protein level of caspase-3, were significantly reinforced, while the anti-apoptotic *Bcl-2* and *XIAP* mRNA levels were markedly attenuated in oocytes exposed to lipopolysaccharide. These results are consistent with a previous report that lipopolysaccharide significantly upregulated mRNA and protein levels of *Bax* and *caspase-3*, and decreased *Bcl-2* expression in MC3T3-E1 cells [40]. Jeon et al. suggested that lipopolysaccharide treatment induced cell death by increasing the level of ROS, which then promoted MAPKs activation and Bax translocation in cultured pneumocytes [41]. Our results indicated that lipopolysaccharide may disrupt oocyte maturation potential by inducing oxidative stress and enhancing the incidence of apoptosis.

Specific epigenetic marks, such as DNA methylation and histone modifications, are critical to confer the female gamete with meiotic as well as developmental competence during gametogenesis [42–44]. DNA methylation is of particular significance in oocyte, as it not only participates in the regulation of gene expression, but also marks specific genes, e.g. imprinted genes in embryo [45]. Incomplete methylation imprints in immature oocytes may be one of the causative factors in inducing developmental abnormalities in mouse [46]. Cheng et al. found that aberrant DNA methylation at specific CpG sites of the suppressors of cytokine signaling 1 promoter correlated with loss of gene expression in lipopolysaccharide-activated macrophages [47]. Several recent studies reported that lipopolysaccharide altered histone lysine methylations, including H3K4me2, H3K9me2 and H3K27me3 in infected cells [48–50]. H3K9me2 is involved in transcriptional repression and the maintenance of a defined expression profile during the final phase of oocyte growth and maturation [51, 52], while H3K4me2 is important for transcriptional activation [53]. SMYD3, a H3K4 methyltransferase, was expressed throughout bovine oocyte *in vitro* maturation and early embryonic development, which suggests that SMYD3 may play an essential role in transcription regulation in bovine [54]. In the current study, lipopolysaccharide significantly decreased the levels of global DNA methylation (5mC) and H3K9me2, whereas the global H3K4me2 levels in oocytes were increased. These results demonstrated that lipopolysaccharide could alter the epigenetic status in bovine oocytes during maturation.

In conclusion, the results of this study suggest that lipopolysaccharide can suppress the first polar body extrusion rate. The negative effects of lipopolysaccharide on maturation potential of bovine oocytes may be partly due to its cytotoxicity on cell cycle progression, meiotic spindle structure, oxidative stress, apoptosis, and epigenetic modifications.

## MATERIALS AND METHODS

### Antibodies and chemicals

Unless otherwise specified, all chemicals were obtained from Sigma-Aldrich Co. (St. Louis, MO, USA). Rabbit polyclonal antibodies for H3K4me2 and H3K9me2 were from Active Motif (Active Motif, CA, USA). Rabbit polyclonal antibody for caspase-3, rabbit monoclonal antibody for p-MAPK and rabbit monoclonal antibody for 5-mC were obtained from Cell Signaling Technology (Beverly, MA, USA). Alexa Fluor 594 goat anti-rabbit secondary antibodies were purchased from Invitrogen (Carlsbad, CA, USA). Annexin V-FITC kits were purchased from Vazyme Biotech Co., Ltd. (Nanjing, China).

### Oocyte *in vitro* maturation (IVM)

The ovaries were harvested from a local abattoir and transported to our laboratory at 28–30°C within 2 h. After washing three times with sterile phosphate-buffered saline (PBS), COCs were manually aspirated from antral follicles that were 2–6 mm in diameter using an 18-gauge needle. Oocytes that were surrounded by at least three compact cumulus layers and had uniform ooplasm were selected. Generally, prior to IVM, COCs were washed three times in IVM medium and 50 COCs were then incubated in 750 μL maturation medium in each well of a four-well plate (Nunc, Denmark) in air with 5% CO_2_ at 38.5°C and the maximum humidity. IVM was carried out in TCM-199 (Gibco BRL, Grand Island, NY, USA) containing 0.01 IU/mL follicle-stimulating hormone (FSH), 10 IU/mL luteinizing hormone (LH), 1 μg/mL estradiol (E2), and 10 % (v/v) fetal bovine serum (FBS; Gibco BRL).

### Lipopolysaccharide treatment

Lipopolysaccharide was dissolved in water and diluted with maturation medium to obtain a final concentrations of 1 μg/mL and 10 μg/mL. The proportion of water in the final culture meidum should not exceed 1% after dilution with *in vitro* maturation medium.

### Cytochemical staining

After culture for 12 h or 22 h, cumulus cells were removed by repeated pipetting. Denuded oocytes were fixed in 3.7% (w/v) PFA in phosphate-buffered saline (PBS) for 1 h, and then permeabilized with PBS supplemented with 1% Triton X-100 for 1h. After washing three times in PBS containing 0.1% polyvinyl alcohol (PVA), the oocytes were blocked in 1% bovine serum albumin (BSA)/PBS for 8 h. The oocytes were incubated with H3K4me2 antibody (1:1000), H3K9me2 antibody (1:1000), p-MAPK antibody (1:200), phalloidin-TRITC (1:500), and anti-α-tubulin-FITC (1:200) antibody overnight at 4°C. After washing three times (5 min each time) in 0.1% PVA/PBS medium, oocytes were labeled with secondary antibodies for 40 min at room temperature and then incubated with 2 μg/mL 4’-6-diamidino-2-phenylindole (DAPI, Roche). Samples from each groups were transferred onto identical slides and analyzed under a confocal microscope (Olympus FV1000, Tokyo, Japan).

For staining of cellular ROS and DHE levels, the samples were incubated with 10 mM 2′,7′-dichlorofluorescin diacetate (DCHFDA) and superoxide-sensitive fluorescent dyes in the dark for 30 min, and then washed three times in washing buffer. Annexin V/PI-FITC kits were used to detect early apoptosis according to the manufacturer's instructions. Samples from each group were transferred onto identical slides and analyzed under a confocal microscope (Olympus FV1000).

For 5 mC staining, the zona pellucid of oocytes was removed by using 0.05% pronase in PBS. All samples were then denatured in 2 N HCl meidum for 30 min and neutralized in 100 mM Tris-HCl medium (pH 8.5) for 10 min. The samples were washed three times in 1% (w/v) BSA/PBS, and then incubated with 1:500 mouse anti-5-mC antibody overnight at 4°C followed by incubation with secondary antibodies for 40 min. Then the samples were washed three times and incubated with 2 μg/mL DAPI. Samples from each group were transferred onto identical slides and analyzed under a laser scanning confocal microscope (Olympus FV1000).

The experiments were repeated three times and at least 10-15 oocytes per group in each replication. Image J software (NIH) was used to analyze the fluorescence intensity, and ROI was used to examine the fluorescence intensity per pixel within the images. Finally, the average values of all images were calculated and used as the final intensities for each group.

### Real-time quantitative PCR analysis

Total RNA was extracted from 100 oocytes treated with or without lipopolysaccharide for 12 h using Trizol reagent (Invitrogen, Carlsbad, CA, USA). First-strand cDNA was synthesized using 0.05 μg of RNA with random hexamers. Real-time quantitative PCR was conducted in three replicates using an ABI-7900 SDS instrument (Applied Biosystems, Foster city, CA, USA) and the primers were listed in Table 1. 2^−ΔΔCT^ method was used to calculate gene expression level with *GAPDH* as a reference gene.

**Table 1:** Primer sequences used for real-time quantitative PCR

Gene	Primer	Sequence (5′-3′)	Annealing temp. (°C)	Fragment size (bp)
*GADPH*	Forward	GGGTCATCATCTCTGCACCT GGTCATAAGTCCCTCCACGA	60	177
	Reverse			
*caspase −3*	Forward	TACTTGGGAAGGTGTGAGAAAACTAA AACCCGTCTCCCTTTATATTGCT	59	71
	Reverse			
*Bax*	Forward	GGCTGGACATTGGACTTCCTTC TGGTCACTGTCTGCCATGTGG	61	112
	Reverse			
*Bcl-2*	Forward	GAGTCGGATCGCAACTTGGA CTCTCGGCTGCTGCATTGT	60	120
	Reverse			
*XIAP*	Forward	GAAGCACGGATCATTACATTTGG CCTTCACCTAAAGCATAAAATCCAG	59	89

### Western blot analysis

150 bovine oocytes at the MI stage were harvested and washed three time in PBS buffer, proteins were extracted and seperated by sodium dodecyl sulfate (SDS)-polyacrylamide gel electrophoresis (PAGE) using 7.5% polyacrylamide gel and tranferred onto membranes (Millipore). Next, the membranes were blocked in 5% (w/v) skim milk overnight at 4°C. Membranes were incubated with p-MAPK monoclonal antibody (1:2000) and caspase-3 polyclonal antibody (1:1000) at 4°C overnight, washed three times (5 min each time) in TBS, and incubated with horseradish peroxidase (HRP)-conjugated secondary antibodies (Zhong Shan Biotechnology, Beijing, China) for 1 h at room temperature. The signals were detected using an ECL kit.

### Statistical analysis

At least three biological replicates were used, and the results were presented as mean ± SEM. Analysis of variance (ANOVA) was used for statistical comparisons and Duncan's multiple comparisons test was used to analyze the differences between each group. The value of *P* < 0.05 was considered statistically significant.
